# Analysis of the response regulatory network of pepper genes under hydrogen peroxide stress

**DOI:** 10.3389/fpls.2022.1018991

**Published:** 2022-12-08

**Authors:** Bingqian Tang, Guangbin Yang, Juan Du, Lingling Xie, Jin Wang, Luzhao Pan, Yin Luo, Qingyun Shan, Xuexiao Zou, Cheng Xiong, Feng Liu

**Affiliations:** ^1^ College of Horticulture, Hunan Agricultural University, Changsha, Hunan, China; ^2^ Longping Branch, Graduate School of Hunan University, Changsha, Hunan, China; ^3^ Engineering Research Center for Germplasm Innovation and New Varieties Breeding of Horticultural Crops, Key Laboratory for Vegetable Biology of Hunan Province, College of Horticulture, Hunan Agricultural University, Changsha, China; ^4^ Laboratory of Lingnan Modern Agriculture, Guangzhou, Guangdong, China

**Keywords:** hydrogen peroxide stress, reactive oxygen species, pepper, gene regulatory network, tissue specificity

## Abstract

Hydrogen peroxide (H_2_O_2_) is a regulatory component related to plant signal transduction. To better understand the genome-wide gene expression response to H_2_O_2_ stress in pepper plants, a regulatory network of H_2_O_2_ stress-gene expression in pepper leaves and roots was constructed in the present study. We collected the normal tissues of leaves and roots of pepper plants after 40 days of H_2_O_2_ treatment and obtained the RNA-seq data of leaves and roots exposed to H_2_O_2_ for 0.5–24 h. By comparing the gene responses of pepper leaves and roots exposed to H_2_O_2_ stress for different time periods, we found that the response in roots reached the peak at 3 h, whereas the response in leaves reached the peak at 24 h after treatment, and the response degree in the roots was higher than that in the leaves. We used all datasets for K-means analysis and network analysis identified the clusters related to stress response and related genes. In addition, *CaEBS1*, *CaRAP2*, and *CabHLH029* were identified through a co-expression analysis and were found to be strongly related to several reactive oxygen species-scavenging enzyme genes; their homologous genes in Arabidopsis showed important functions in response to hypoxia or iron uptake. This study provides a theoretical basis for determining the dynamic response process of pepper plants to H_2_O_2_ stress in leaves and roots, as well as for determining the critical time and the molecular mechanism of H_2_O_2_ stress response in leaves and roots. The candidate transcription factors identified in this study can be used as a reference for further experimental verification.

## Introduction

Biotic and abiotic stresses are the main environmental factors that restrict normal growth and development of plants by decreasing the yield, thereby posing a threat to food safety (Fedoroff et al., 2010; Zhu, 2016).

Hydrogen peroxide (H_2_O_2_), an important regulatory component of plant signal transduction, affects plant growth in a concentration-dependent manner (Xiong et al., 2015). In a root dipping treatment of *Solanum lycopersicum*, low doses of H_2_O_2_ (0.1 or 0.5 mM) were reported to be potentially advantageous for the development of plants (Nazir et al., 2019). In a study, the dose of 0.1 mM H_2_O_2_ was found to be optimum as it caused a maximum increase in the length, fresh mass, and dry mass of *Solanum lycopersicum* roots and shoots (Nazir et al., 2019). In cucumbers, a low concentration of H_2_O_2_ improved the germination capability, germination rate, tap root length, and hypocotyledonary axis, whereas high H_2_O_2_ concentration inhibited these processes (Sun et al., 2009). On the one hand, the production of reactive oxygen species (ROS) is a byproduct of aerobic metabolism (Sandalio and Romero-Puertas, 2015; Dietz et al., 2016; Huang et al., 2016). On the other hand, anaerobic metabolism can be induced in plants under biotic or abiotic stress including pathogens, strong light, drought, low temperature, high temperature, and salinization (He et al., 2018b). Different ROS such as H_2_O_2_, singlet oxygen, and superoxide can trigger specific changes in a transcriptome (Gadjev et al., 2006; Willems et al., 2016).

The biological effects of ROS on cell cytotoxicity or signaling mainly depend on the balance between generating and scavenging systems. The normal concentration of ROS plays a crucial role in maintaining normal plant growth and improving plant stress resistance (Karuppanapandian et al., 2011; Huang et al., 2019).

High ROS concentrations hinder the growth and development of plants and even lead to plant death, thereby causing an irreversible damage to plants (Sachdev et al., 2021). To cope with the ever-changing external environment, plants have evolved a complex mechanism for excessive ROS scavenging (Huang et al., 2019). The scavenging mechanism of ROS in plants can be divided into two types. First, the enzymatic scavenging mechanism, which mainly includes superoxide dismutase (SOD), ascorbate peroxidase (APX), glutathione peroxidase (GPX), catalase (CAT), and glutathione S-transferase. Second, the non-enzymatic scavenging mechanism, which mainly depends on ascorbic acid, reduced glutathione, α-tocopherol, proline, alkaloids (carotenoids), and flavonoids (Gill and Tuteja, 2010; Miller et al., 2010; Sarvajeet Singh Gill et al., 2011; Das and Roychoudhury, 2014).

ROS also affect some transcription factors such as HSF, NPR1, WRKY, and MYB. The upregulation of the expression of these transcription factor genes eventually leads to changes in the expression of downstream genes. Gene expression changes triggered by ROS are mediated by stress-responsive cis-regulated promoter elements, redox regulation of transcription regulators, and upstream signal cascades such as the mitogen-activated kinase module (Dietz, 2014; He et al., 2018a).

The present study investigated changes in whole-genome expression in hot peppers under H_2_O_2_ stress and determined the ROS-scavenging gene response mechanism at the gene level. Data were obtained from the transcriptome data of leaves and roots of peppers cultured in water for 40 days with 30 mM H_2_O_2_ at six time points and compared with those of the control group. A regulatory network of pepper genes that respond to H_2_O_2_ stress was constructed. In addition, the genes related to key ROS-scavenging enzymes in peppers, important time nodes of the response, and the key transcription factors co-expressing with the key enzymes were identified.

## Materials and methods

### Plant materials and data sources

Total RNA-seq data from H_2_O_2_-treated pepper leaves and roots was isolated according to the method described in a previous study (Liu et al., 2017). Line 6421, an elite-breeding pepper (*Capsicum annuum*) line, was selected from a long-red pepper landrace that is widely grown on the west side of Xiangjiang River, Hunan Province, China.

The seeds were disinfected with sodium hypochlorite and grown in vermiculite at 25/18°C under short-day growth conditions (8 h light/16 h dark, light intensity of 6000 Lux, pH 6.0) and 60%–70% humidity. The stress treatments were applied on 40-day-old seedlings, and samples for RNA extraction were collected from the leaves and roots of the seedlings. The leaves and roots were treated with 30 mM H_2_O_2_ for 0.5, 1, 3, 6, 12, and 24 h to induce oxidative stress. Control plants were mock treated with the nutrient solution only. Leaf and root tissues were collected from both treated and control plants at 0.5, 1, 3, 6, 12, and 24 h after treatment.

### Differential gene expression analysis

We quality-tested gene sequences by using (Chen et al., 2018) FastQC v. 0.11.7 (Andrews, 2017) with default mapping parameters (10 mismatches/read; nine multimapping locations/read) and aligned using HISAT (Kim et al., 2015). Differential gene expression analysis was performed using DESeq2 v1.20.0, an R-based package available from Bioconductor (Love et al., 2014). The reference genome of *C. annuum L.* (Zunla_1) was used in this analysis (Qin et al., 2014).

Transcripts expressing differentially under two conditions were identified by examining the difference in their abundance under these conditions. The abundance of a transcript was measured as the mean-normalized count of reads mapping onto the transcript (Love et al., 2014). The difference in the expression was quantified using the logarithm (the logarithmic multiple change) of the mean-normalized count ratio between the two conditions. The differentially expressed transcripts in our experiments were defined as those with adjusted P values < 0.05 fold changes (FC) (negative binomial Wald test and Benjamini-Hochberg correction; both are part of the DESeq2 package). Differentially expressed genes (DEGs) were classified as upregulated or downregulated according to the significant positive or negative logarithmic change value. upregulated and downregulated DEGs were represented using the Venn diagram (Lin et al., 2016). Heat maps were generated using the seaborn heatmap available in python. Summary statistics for the sequencing performed are presented in **Table S1** and all expression genes in **Table S2**.

### Co-expression cluster identification and gene enrichment analysis

Co-expression/coregulation analysis was performed on the samples from 12 control tissues and 12 H_2_O_2_-treated tissues by using the K-means (Gasch and Eisen, 2002) method (Gasch and Eisen, 2002) in python. The normalized expression values of genes were calculated by dividing their expression values in all samples with their maximum observed TPM (Transcripts Per Kilobase Million) Hierarchical clustering (HCL) and principal component analysis (PCA) were performed using the Kernel Principal Component Analysis in python with default settings to facilitate the graphical interpretation of the relatedness among the 24 samples. The transformed- and normalized-gene and -metabolite expression values with Z-scores were used for HCL and PCA. The clusters that were identified to respond to H_2_O_2_ stress were followed up for gene enrichment analysis with GOATOOLS (Klopfenstein et al., 2018), which is a python package for Gene Ontology (GO) enrichment analysis; false discovery rate (FDR) values of <0.01 was considered to denote statistically significant GO terms. The Kyoto Encyclopedia of Genes and Genomes (KEGG) pathway analysis was performed by KofamKOALA (Aramaki et al., 2020) to protein sequences by homology search, and the enrichment analysis use P-value Cutoff = 0.05 was carried by package clusterProfiler (Yu et al., 2012).

### Analysis of orthologous genes and homologous evolutionary genes

The *Arabidopsis thaliana* (TAIR10) and *C. annuum L.* (Zunla_1) proteomes were downloaded from The Arabidopsis Information Resource and National Center for Biotechnology Information, respectively. Protein sequences from the primary transcripts were used for the construction of orthogroups between the two species by using OrthoMCL (https://orthomcl.org/orthomcl/). The information on transcription factors (TFs) was retrieved from PlantTFDB (http://planttfdb.gao-lab.org/).

The multiple sequence alignment of capsicum and *Arabidopsis* genes was performed using MUSCLE (Multiple Protein Sequence Alignment) in Linux. A contiguous algorithm was used to build phylogenetic tree files by using treebest software (Edgar, 2004), and a phylogenetic tree was constructed using the online tool iTOL (Interactive Tree Of Life; https://itol.embl.de/) (Letunic and Bork, 2016).

### Network analysis of ROS scavenging genes in pepper

The Python NetworkX package (https://networkx.org/) was used to visualize the co-expression network relationship between the candidate target transcription factors and ROS-scavenging enzyme-related genes.

## Results

### Generation of the pepper H_2_O_2_ dataset

**Figure 1:**
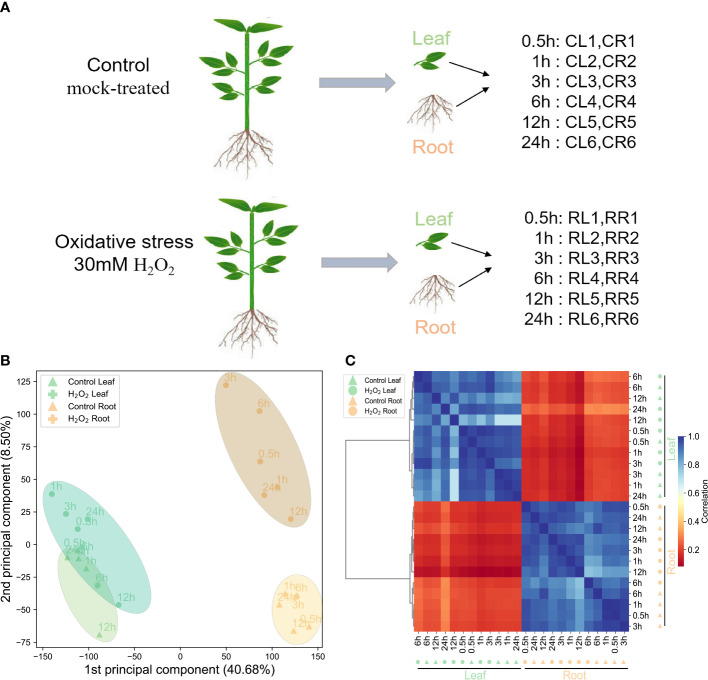
Summary of the transcriptome data of the H_2_O_2_ datasets. **(A)**: Schematic representation of the design for the datasets. Leaf (L) and root (R) of these samples at six time points in the control and H_2_O_2_-treatment groups, respectively. **(B)**: Principal component analysis of the transcriptome data from the control and H_2_O_2_-treatment samples (leaf and root). **(C)**: The hierarchical clustering analysis of the expression profiles of 35,336 genes from the 24 samples; the color scale 0–1 represents the Pearson’s correlation coefficient.

To comprehensively and accurately record the gene response network in leaves and roots of pepper plants treated with H_2_O_2_, we used the transcriptome data set (**Figure 1A**). To determine the gene expression in the 24 samples, a Z-score normalized expression heat map (**Figure 1C**) was obtained. The Pearson’s correlation matrix among the transcriptome samples showed that the gene expression pattern was tissue-specific. The same tissues clustered together (**Figure 1C**). PCA (**Figure 1B**) also showed that the samples from the same tissues gathered at different time points during stress treatment (**Figure 1B**). These results showed that the difference in gene expression because of H_2_O_2_ stress is much smaller than the tissue-specific expression of the whole gene of the pepper roots and leaves, which is consistent with our general cognition.

### The pepper transcriptomes are coregulated in 12 clusters that corresponded to different tissues and treatment times

**Figure 2:**
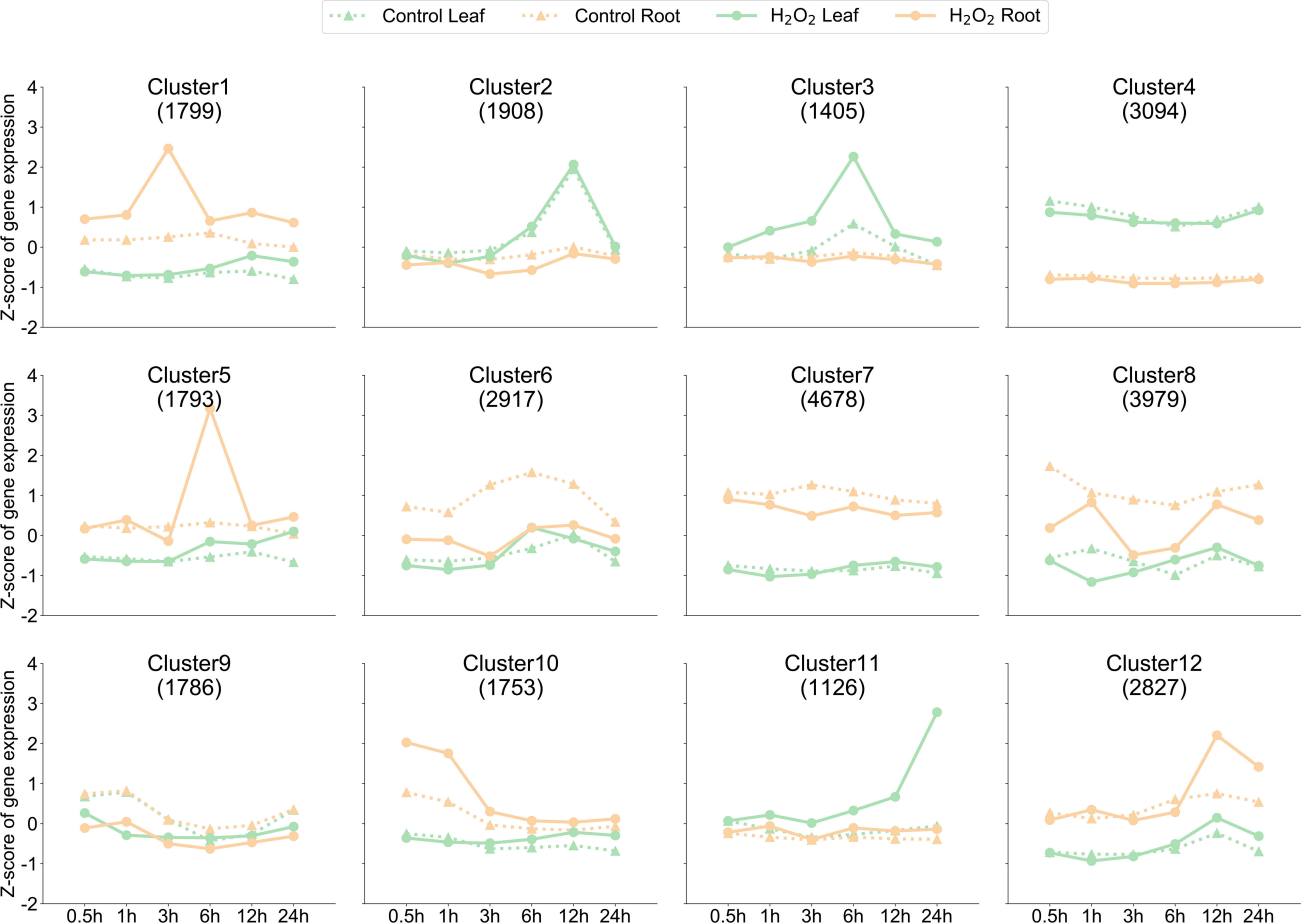
Dynamics of gene expression in the samples. K-means clustering grouped the expression profiles of the transcriptome into 12 clusters. The x-axis depicts the 24 samples, and the y-axis depicts the Z-score standardized cluster gene expression trend. The numbers shown in each box (for example, 1,799 genes for Cluster 1) were derived from the number of genes.

To further understand the trend in the classification of gene expression in pepper roots and leaves after H_2_O_2_ stress, we used the K-means clustering algorithm to divide the genes into 12 clusters according to the expression patterns of all 29,065 genes expressed in at least one sample (**Figure 2**). The expressions of genes in Cluster 1, Cluster 5, and Cluster 12 were significantly increased in the roots at 3 h, 6 h, and 12 h after the stress, respectively, whereas the expressions of genes in Cluster 3 and Cluster 11 were significantly increased in the leaves at 6 h and 24 h, respectively, after the stress. In the early stage of the stress treatment, we observed that the genes in Cluster 10 were the first to respond within 0.5 h–1 h. Despite the increase or decrease in gene expression observed after the stress treatment at a certain time point, genes in Cluster 2, Cluster 4, Cluster 7, and Cluster 9 showed no significant changes in expression in the leaves and roots at the six time points before and after the stress treatment. The reason for dividing the genes into 12 clusters was to classify these genes according to dynamic changes in them to facilitate their follow-up and better targeted analysis.

### Identification of differentially expressed genes under hydrogen peroxide stress

To capture a comprehensive overview of the response, H_2_O_2_-responsive genes in the peppers were identified by analyzing the temporal changes in the transcriptome after up to 24 h of treatment with 30 mM H_2_O_2_ (**Figure 1A**). Overall, 1,116 upregulated and 309 downregulated genes were identified in the leaves, whereas 1,144 upregulated and 394 downregulated genes were identified in the roots at different time points. The analyses collectively yielded 2,382 DEGs, constituting approximately 8.20% of the expressed genes in the datasets.

**Figure 3:**
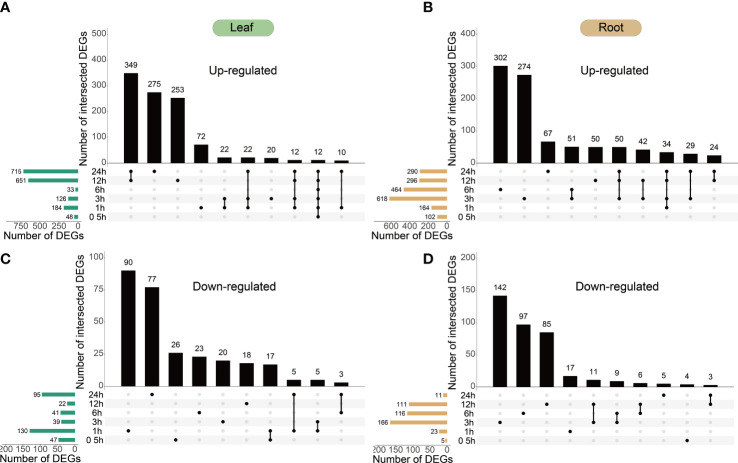
Temporal dynamics of *Capsicum annuum L.* transcriptome during H_2_O_2_ treatment. **(A–D)**, the 40-day-old *C. annuum L.* plants were treated with 30 mM H_2_O_2_ in liquid media and harvested at the given time points for transcriptome analysis. UpSet plots of the number of upregulated and downregulated genes (cut-off threshold, |log2(FC)| ≥2; FDR <0.01) demonstrating different temporal expression patterns (top bar graphs). The total number of upregulated and downregulated genes at each time point is shown on the left. All leaf and root DEGs show in **Supplementary Table S3**.

The difference in differential expression between the leaves and roots was that the highest amount of DEGs in the leaves was observed at 12 and 24 h (**Figures 3A, C**) and that in the roots was observed at 3 and 6 h (**Figures 3B, D**); in leaves, 349 DEGs were upregulated and 17 DEGs were downregulated at 12 and 24 h. Consequently, the gene response speed in the roots was faster than that in the leaves of peppers under H_2_O_2_ stress. The distribution of upregulated and downregulated DEGs identified in the leaves and roots in each cluster is shown in **Table 1**, and the results of the distribution corresponding to the trend map are shown in **Figure 2**.

**Table 1:** Distribution of the leaf and root DEGs identified in this study across different cluster.

	Cluster												
	1	2	3	4	5	6	7	8	9	10	11	12
Genes	1799	1908	1405	3094	1793	2917	4678	3979	1786	1753	1126	2827	Number
Leaf Upregulated DEGs	213	36	116	41	94	19	91	9	3	139	135	220	1116
Leaf Downregulated DEGs	30	14	2	22	5	24	93	33	46	21	1	18	309
Root Upregulated DEGs	297	41	46	93	222	0	8	1	22	140	101	173	1144
Root Downregulated DEGs	0	30	11	112	5	90	58	39	36	2	6	5	394

### Functional enrichment analysis of DEGs

**Figure 4:**
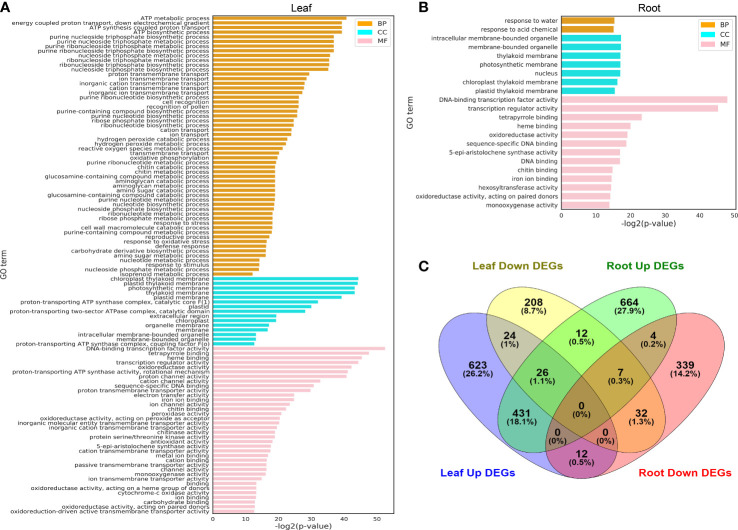
GO terms of leaf and root DEGs under hydrogen peroxide stress. **(A)** GO terms identified from the different treatment time points of leaf DEGs (FDR < 0.01). **(B)**: GO terms identified from the different treatment time points of root DEGs (FDR < 0.01). **(C)**: Venn diagrams of upregulated and downregulated DEGs identified in the leaf and root tissues. The full lists of DEGs and GO terms from the enrichment analysis are shown in **Supplementary Tables S4, S5**.

The main functions of DEGs (shown in **Figure 3**) identified in the leaf (**Figure 4A**) and root (**Figure 4B**) were analyzed by GO, and the aggregation of upregulated DEGs and downregulated DEGs in the leaf and root is shown by the Wayne diagram (**Figure 4C**). Most identified DEGs encode the enzymes involved in basic biological metabolism and nucleotide binding (**Figures 4A, B**), and the amount of GO terms enriched by DEGs in leaves was more than that in roots.

Several key points identified in GO term enrichment in the leaf and root DEGs were related to the oxidoreductase activity, which implied that the H_2_O_2_ treatment of pepper plants affected key physiological metabolic pathways *in vivo*. In the leaves, GO terms such as “ATP metabolic process”, “chloroplast thylakoid membrane”, “proton-transporting ATP synthase activity, rotational mechanism” and “oxidoreductase activity, acting on peroxide as acceptor”, leaf photosynthesis, and energy transformation were closely related; in roots, GO terms such as “response to water”, “intracellular membrane-bounded organelle,” “DNA-binding transcription factor activity,” and “oxidoreductase activity” were also related to cell redox and important organelle membranes.

**Figure 5:**
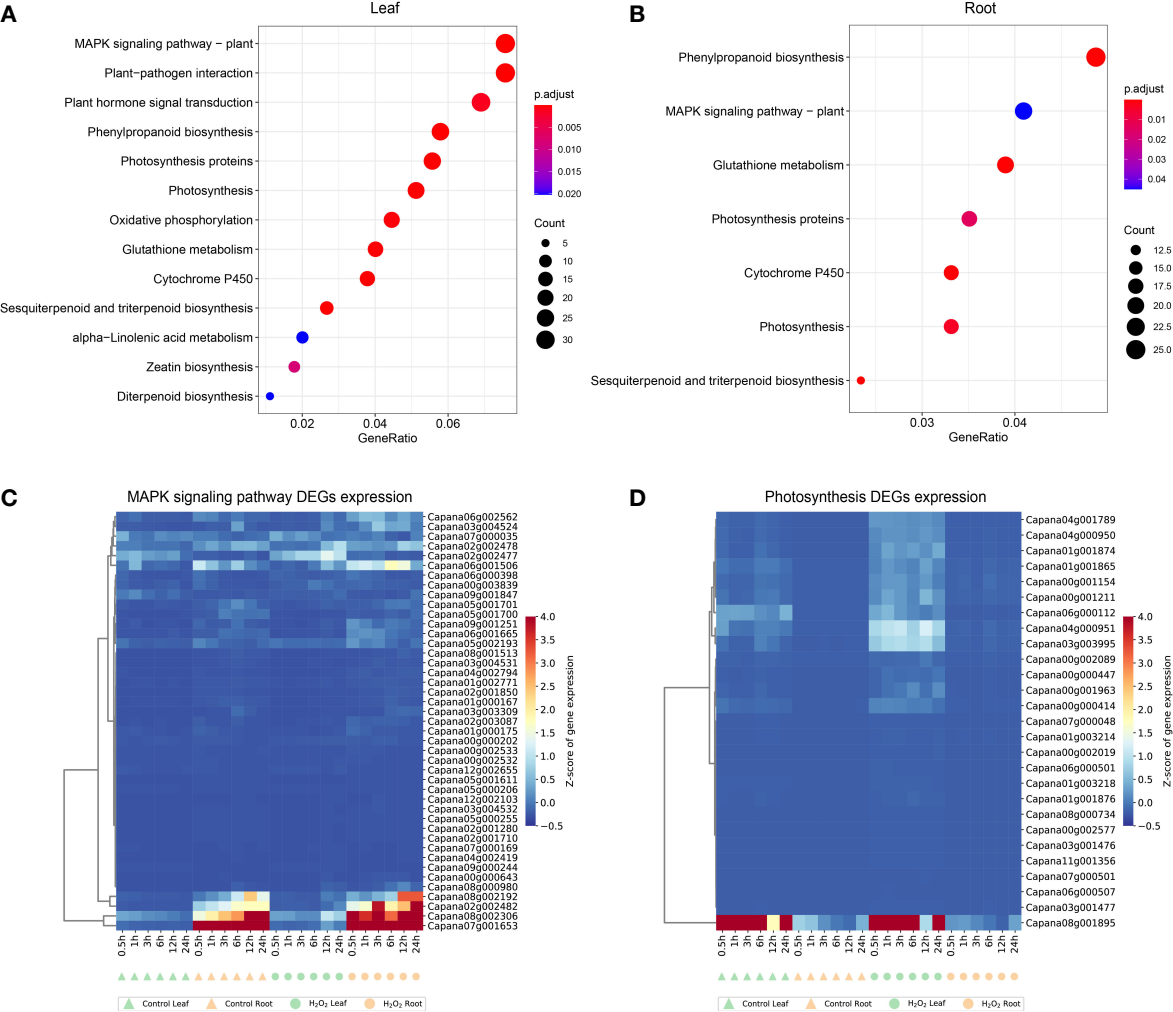
Kyoto encyclopedia of genes and genomes (KEGG) pathway enrichment analysis results for the DEGs in leaf **(A)** and root **(B)**. Expression profiles of DEGs enriched in MAPK-signaling pathway **(C)** and photosynthesis pathway **(D)** in leaves and roots. The complete list of DEGs and KEGG pathways for enrichment analysis is shown in **Supplementary Tables S6, S7**.

Through the KEGG pathway analysis of the DEGs identified in leaf and root at 6 time points, it was found that the common enrichment in “MAPK signaling pathway”, “Phenylpropanoid biosynthesis”, “Photosynthesis”, “Glutathione metabolism”, “Cytochrome P450” and “Sesquiterpenoid and triterpenoid biosynthesis” (**Figure 5**). However, “Plant hormone signal transduction”, “Oxidative phosphorylation”, “alpha−Linolenic acid metabolism”, “Zeatin biosynthesis” and “Diterpenoid biosynthesis” were unique in leaf (**Figure 5A**).

We selected 43 and 27 genes in the MAPK-signaling pathway (ko04016) and photosynthesis pathway (ko00195), respectively, those genes were enriched in both the DEGs identified in leaves and roots (**Figures 5C, D**). In plants, several MAPK-signaling pathways are triggered by different biotic and abiotic stress stimuli, such as pathogen infection, injury, low temperature, drought, osmotic shock, high salinity, and reactive oxygen species (Asai et al., 2002; Colcombet and Hirt, 2008; Rodriguez et al., 2010). The Photosynthesis pathway is an important route for energy conversion in plants (Jones and Fyfe, 2001; Ferreira et al., 2004). DEGs identified in leaves and roots are enriched in these two pathways, indicating that the fluctuation of gene expression in response to external stimuli is conserved to a certain extent in different tissues, and the genes shown in **Figures 5C, D** are important candidate genes.

### Evolution and expression analysis of genes associated with ROS scavenging

**Figure 6:**
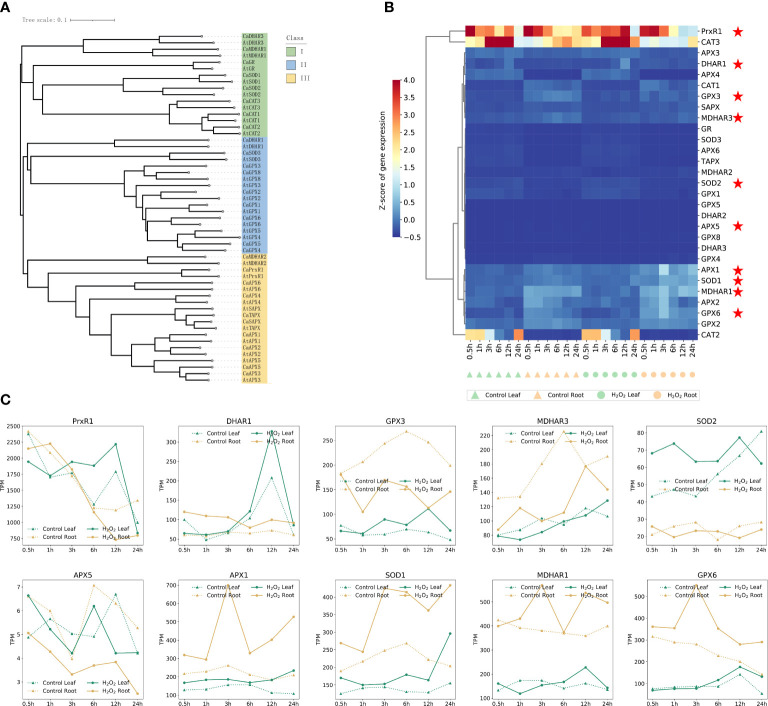
The genetic relationship of enzymes related to ROS scavenging in *Arabidopsis thaliana* and capsicum **(A)**, and the gene expression patterns in Pepper **(B)**. All those pepper genes expression in **Supplementary Table S8**. **(C)** Expression profiles of 10 typical ROS-scavenging enzyme synthesis genes in different clustering modules at different time points in leaves and roots of control and H_2_O_2_ stress.

Because of the increase in the ROS content in plants observed under H_2_O_2_ stress, we evaluated the response of the genes associated with ROS scavenging. The experiment was designed to evaluate changes in the expression patterns of ROS-scavenging genes such as SOD, APX, GPX, and CAT that are involved in the enzymatic scavenging mechanism (Gill and Tuteja, 2010; Miller et al., 2010; Sarvajeet Singh Gill et al., 2011). Additionally, we determined whether these genes have different response nodes and levels in the leaves and roots, according to the sequence of these genes in *A. thaliana*. The respective lineal homologous genes were identified in the pepper genome; 28 genes synthesizing these key enzymes in *A. thaliana* and 28 lineal homologous genes in pepper were identified. The evolutionary relationship of these 58 genes is shown in **Figure 5A**, which were mainly divided into three subfamilies, and a close sequence alignment relationship between *A. thaliana* genes and the corresponding pepper lineal homologous genes was obvious. The results reflected the significance of identifying direct homologous genes. Subfamily I was mainly from *A. thaliana* and capsicum and included *DHAR3*, *MDHAR1*, *GR*, *SOD1/2*, and *CAT2*. Subfamily II mainly included *MDHAR2* and *PrxR1*. All *APXs*, *DHAR1*, *SOD3*, and all *GPXs* were included in subfamily III (**Figure 6A**). **Figure 3B** shows the expression patterns of the genes encoding for these ROS-scavenging enzymes in untreated leaves and roots, as well as in the treated leaves and roots. *CAT3* expression in root tissues under H_2_O_2_ stress was significantly decreased compared with that in untreated roots; *PrxR1* gene expression in root tissues after12 h and 24 h was also decreased after root treatment (**Figure 6B**).

However, the expression of six genes, namely *APX1, SOD1, MDHAR1, APX2, GPX6*, and *GPX2*, was higher at 3 h after root treatment than that in the untreated root tissues; however, no significant difference was observed in the gene expression across other time periods and tissues. These six genes may be important in response to ROS clearance in the roots treated for 3 h. Follow-up studies on H_2_O_2_ stress must focus on the expression of genes in roots at 3 h, which can also be seen from **Figures 3B, D**. The number of DEGs in the roots treated with H_2_O_2_ for 3 h was the highest, and among these genes, 618 genes were upregulated genes and 196 genes were downregulated. The effect of H_2_O_2_ stress on plant roots reached a peak at 3 h compared with that in the control, and most of the DEGs were expressed in the roots.

According to the expression profiles of 29 genes related to ROS-scavenging enzyme synthesis at different time points in the control and H_2_O_2_ stress of leaves and roots shown in **Figure 6B**, 10 obvious genes are marked on the basis of different expression patterns after H_2_O_2_ stress. The genes whose expression levels changed and the expression profiles of these 10 genes are shown separately to avoid the influence of data normalization in **Figure 6C**. *PrxR1*, *DHAR1*, *GPX3*, *SOD2*, and *SOD1* were significantly up-regulated at different time points in leaves under hydrogen peroxide stress; *APX1*, *SOD1*, *MDHAR1*, and *GPX6* were up-regulated in roots after H_2_O_2_ stress, which may be related to the fact that the roles of these enzymes in pepper were tissue related.

### Discovery and functional analysis of transcription factors of important regulatory genes responding to H_2_O_2_ stress and the analysis of key clusters

Several key clusters identified through the K-means cluster analysis may have a certain role in response to ROS clearance. Most genes showed a rapid increase in the expression in leaves or roots at a certain time point, which may be because of plant response to irreversible damage caused by an increase in the ROS level, and the study of genes in these key clusters can be helpful in determining the response mechanism of plants to H_2_O_2_ stress. Moreover, these clusters have an increase in expression in different tissues at different time points, indicating an important area of research for determining time nodes and patterns of gene response to ROS. For instance, the genes in Cluster 10 (1,753 genes) were the first to respond to H_2_O_2_ stress in root tissues. Within 0.5 and 1 h, the expression of genes in Cluster 10 in roots under H_2_O_2_ stress was much higher than that in untreated roots; however, the difference in the expression of these genes was not significant in the leaves during the same period. The expression of genes in Cluster 1 (1,799 genes) increased significantly in roots under 3-h stress, and at this time point, the largest number of DEGs was observed in the roots (**Figures 3B, D**), indicating that the 3-h time point is the most favorable time point for gene response to stress in pepper roots.

To further study the function of genes in Cluster 1 and Cluster 10, we performed GO enrichment analysis of all genes in these clusters. A total of 1,799 genes in Cluster 1 were mainly enriched in the GO terms such as “L-phenylalanine metabolic process”, “DNA-binding transcription factor activity,” and “acyltransferase activity”. GO terms enriched by 1,753 genes in Cluster 10 were “oligosaccharide metabolic process”, “protein serine/threonine kinase activity”, “kinase activity,” and “ubiquitin-protein transferase activity”.

**Figure 7:**
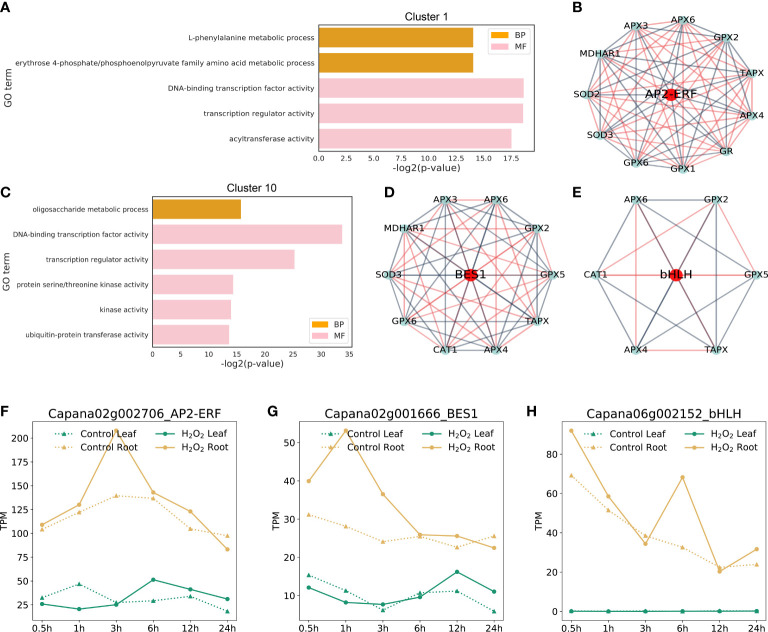
Important clusters in GO enrichment and TF networks. **(A, C)**: GO terms of the identified DEGs from Cluster 1 and Cluster 10 (FDR < 0.01)). The complete lists of cluster genes and GO terms from the enrichment analysis are shown in **Supplementary Tables S9, S10**. **(B, D, E)**: The transcriptional factors that co-expressed with ROS-scavenging genes were identified using the constructed network. **(F–H)**: Expression profiles of the three candidate transcription factors at different time points in control and H_2_O_2_ stress in leaves and roots.

By analyzing the constructed gene co-expression relationship network, we found that the correlation coefficient of key transcription factors in Cluster 1 and Cluster 10 was closely related to those of ROS-scavenging genes. The relationship between these three typical transcription factors and ROS-scavenging enzyme-related genes (**Figure 7B**) is shown in the form of a network map (**Figures 7D, E**). The genes in the middle red circle represent the key transcription factors that were identified. The genes in the blue circle on the edge are the genes related to ROS-scavenging enzymes in capsicum. The blue lines between the two genes indicate negative regulatory relationships, whereas the red lines indicate positive regulatory relationships. These three key transcription factors were verified as the key factors in subsequent experiments, providing a new conjecture for studying the molecular mechanism of ROS scavenging in plants.

Through sequence alignment, the corresponding orthologous genes of three transcription factors, namely *BES1*, *RAP2* and *bHLH029*, in Arabidopsis were identified, and the sequence similarity was more than 70%. Therefore, we named the corresponding pepper genes as *CaBES1* (**Figures 7B, F**), *CaRAP2* (**Figures 7D, G**), and *CabHLH029* (**Figures 7E, H**) according to the gene names of Arabidopsis thaliana. The gene expression profile data of these were extracted. These data are displayed as line graphs in **Figures 7F–H**. The expression levels of *CaBES1* and *CabHLH029* were significantly up-regulated at H_2_O_2_ stress 3 and 6 h, respectively, but there was no significant difference at other time points. *CaRAP2* was significantly up-regulated in the early stage of H_2_O_2_ stress (0.5–3 h), and its expression returned to normal in the later stage of H_2_O_2_ stress. They may be involved in regulation at different stages.

## Discussion

Pepper is an important vegetable belonging to the Solanaceae family that is widely cultivated worldwide. According to FAO statistics (www.fao.org), peppers were grown in 118 countries and regions of the world in 2018. Pepper is the third largest vegetable crop in the world; in 2014, Mexico and China successively completed the whole-genome sequencing of two pepper varieties, namely CM334 and Zunla-1 (Kim et al., 2014; Qin et al., 2014). The pepper genome sequence is ∼3.3 Gb, which is approximately three times that of the tomato genome (∼900 Mb) (Tomato Genome, 2012). Because of the presence of many tandem repeats in the pepper genome, the research on pepper gene annotation is relatively lagging. The present study analyzed the RNA-seq data of the leaves and roots of 40-day-old pepper plants under H_2_O_2_ stress at six time points and compared the obtained data with those of the control plant, we used data from this database (Liu et al., 2017). Moreover, the study analyzed the dynamic expression network of genes in leaves and roots in response to H_2_O_2_ stress from 0.5 h to 24 h at the genomic level. Based on the gene expression trend, the K-means value was used to divide genes into 12 expression clusters (**Figure 2**) from Cluster 1 to Cluster 10. The GO enrichment of genes in the clusters was analyzed (**Figures 7A, C**), and the genes in the chili peppers were identified through the orthologous alignment of the determined gene sequences in *Arabidopsis*. A total of 28 synthesis genes related to ROS-scavenging enzymes were identified, which showed their dynamic expression at six time points under H_2_O_2_ stress. We constructed a co-expression network matrix based on the Pearson correlation coefficient and found three transcription factors that exhibited a strong correlation with the synthesis genes of multiple ROS-scavenging enzymes (**Figures 4C–F**). Subsequent experiments are required to study in detail the mechanism through which these transcription factors regulate the associated enzymes when plants are under H_2_O_2_ stress, as well as the mechanism through which plants balance the ROS concentration.

The pepper plants in the present study are based on hydroponic culture and 30 mM H_2_O_2_ treatment in the aqueous solution. The roots most directly sense the changes in ROS concentrations, whereas the leaves after the stress. The response to H_2_O_2_ stress was higher and the response time was earlier in pepper roots than in leaves (**Figure 3**). After H_2_O_2_ stress, it reached to genes in pepper roots within 3 h, denoting the peak of the response (**Figures 3B, D**) and indicating that the number of DEGs was the largest at 3 h. Unlike the response speed in the roots, the peak of gene response in the leaves appeared 24 h after H_2_O_2_ stress (**Figures 3A, C**). Relative to roots, the leaves responded gradually, and previous studies have shown that ROS play a crucial role in controlling root hair growth (Carol and Dolan, 2006). Some studies have used large quantities of transcriptome data processed by *Arabidopsis* oxidative stress and found that the time of oxidative stress is the determinant of the footprint of different transcriptomes (Gadjev et al., 2006; Willems et al., 2016). Thus, exploring the differences in gene expression in plants at different time points after oxidative stress treatment can be valuable.

Through the cluster analysis of identified genes related to ROS clearance and key responses in peppers, the key transcription factors, including *CaARP2* (**Figure 7B**) in Cluster 10 and *CaBES1* and *CabHLH029* in Cluster 1, were identified (**Figures 7D, E**). These transcription factors were co-expressed with the genes encoding for multiple ROS-scavenging enzymes, and their K-means analysis results showed their distribution in Cluster 1 and Cluster 10. The genes in Cluster 10 showed the earliest response in pepper root tissues. The genes in Cluster 1 had the largest number of DEGs at the 3-h time point in the root tissues of pepper under H_2_O_2_ stress compared with the unstressed root tissues. Therefore, these Clusters 1 and 10 were used to analyze the response of genes in pepper roots to peroxidation. The co-expression of the three transcription factors and multiple genes encoding for ROS-scavenging enzymes provides an important reference for the subsequent verification of their functional mechanisms. Studies on *Arabidopsis* have confirmed that *BES1* encodes for brassinosteroid-signaling (BRs) protein that accumulates in the nucleus in the dephosphorylated form in response to BRs and coordination of chloroplast development (Wang et al., 2002; Yin et al., 2005; Zubo et al., 2018; Clark et al., 2021; Van Nguyen et al., 2021) in *A. thaliana*, which is in line with our results that *CaBES1* also affected the expression of genes in response to H_2_O_2_ stress in pepper, thus indicating that the aforementioned mechanism may also be involved in pepper. For Arabidopsis, many studies have shown that *RAP2* is involved in oxygen sensing, which plays a key role in controlling root bending in response to hypoxia (Hartman et al., 2019; Smit et al., 2020; Tang et al., 2021; Seok et al., 2022); *bHLH029*, as a putative transcription factor encoding the regulation of iron uptake responses, mRNA was detected in the outer cell layer of roots and accumulated in iron deficiency, similar to FER in tomato, as a regulator of iron uptake (Cai et al., 2021; Kanwar et al., 2021; Song et al., 2022). The functions of these three important transcription factors of pepper identified in the gene expression network of pepper seedlings under hydrogen peroxide stress may show some functional conservation with their homologous genes in Arabidopsis, but to obtain exact evidence further experiments are required.

In this study, based on the control and processed RNA-seq data of the leaves and roots of 40-day-old pepper plants subjected to H_2_O_2_ stress at six time points, the genome-wide regulatory network was analyzed, and three possible key transcription factors were identified. A balanced candidate transcription factor can provide reliable evidence for in-depth verification and analysis.

## Conclusion

In this study, the gene dynamic expression network of pepper leaves and roots at six time points in response to H_2_O_2_ stress was analyzed at the whole-genome level. A total of 29,065 genes were found to be expressed in at least one sample. These genes were divided into 12 clusters based on their dynamic expression in different tissues and time points. In addition, based on the research on ROS-scavenging enzymes in *Arabidopsis*, we identified 28 orthologous genes in peppers that were associated with Cluster 1 and 10 and identified three transcription factors. Multiple ROS-scavenging enzyme synthesis-related genes in pepper were highly co-expressed. We also performed GO and KEGG analysis for the DEGs identified in leaves and roots to identify key cluster genes and analyzed the GO terms and KEGG pathways that are mainly enriched in these important genes. This study provides insights into the effects of H_2_O_2_ stress on pepper at the whole-genome level and may serve as a reference for research on other crops.

## Data Availability

The datasets presented in this study can be found in online repositories. The names of the repository/repositories and accession number(s) can be found below: China National Center for Bioinformation, accession number CRA007961.

## References

[B1] Andrews S. (2017). FastQC: a quality control tool for high throughput sequence data. 2010. doi: 10.12688/f1000research.21142.2

[B2] Aramaki T. Blanc-Mathieu R. Endo H. Ohkubo K. Kanehisa M. Goto S.. (2020). KofamKOALA: KEGG ortholog assignment based on profile HMM and adaptive score threshold. Bioinformatics 36, 2251–2252. doi: 10.1093/bioinformatics/btz859 31742321 PMC7141845

[B46] Asai T. Tena G. Plotnikova J. Willmann M. R. Chiu W.-L. Gomez-Gomez L.. (2002). MAP kinase signalling cascade in arabidopsis innate immunity. Nature 415 (6875), 977–983. doi: 10.1038/415977a 11875555

[B4] Cai Y. Li Y. Liang G. (2021). FIT and bHLH ib transcription factors modulate iron and copper crosstalk in arabidopsis. Plant Cell Environ. 44, 1679–1691. doi: 10.1111/pce.14000 33464620

[B5] Carol R. J. Dolan L. (2006). The role of reactive oxygen species in cell growth: Lessons from root hairs. J. Exp. Bot. 57, 1829–1834. doi: 10.1093/jxb/erj201 16720604

[B6] Chen S. Zhou Y. Chen Y. Gu J. (2018). Fastp: an ultra-fast all-in-one FASTQ preprocessor. Bioinformatics 34, i884–i890. doi: 10.1093/bioinformatics/bty560 30423086 PMC6129281

[B7] Clark N. M. Nolan T. M. Wang P. Song G. Montes C. Valentine C. T.. (2021). Integrated omics networks reveal the temporal signaling events of brassinosteroid response in arabidopsis. Nat. Commun. 12, 5858. doi: 10.1038/s41467-021-26165-3 34615886 PMC8494934

[B8] Colcombet J. Hirt H. (2008). Arabidopsis MAPKs: A complex signalling network involved in multiple biological processes. Biochem. J. 413, 217–226. doi: 10.1042/BJ20080625 18570633

[B9] Das K. Roychoudhury A. (2014). Reactive oxygen species (ROS) and response of antioxidants as ROS-scavengers during environmental stress in plants. Front. Environ. Sci. 2. doi: 10.3389/fenvs.2014.00053

[B10] Dietz K. J. (2014). Redox regulation of transcription factors in plant stress acclimation and development. Antioxid Redox Signal 21, 1356–1372. doi: 10.1089/ars.2013.5672 24182193

[B11] Dietz K. J. Turkan I. Krieger-Liszkay A. (2016). Redox- and reactive oxygen species-dependent signaling into and out of the photosynthesizing chloroplast. Plant Physiol. 171, 1541–1550. doi: 10.1104/pp.16.00375 27255485 PMC4936569

[B12] Edgar R. C. (2004). MUSCLE: multiple sequence alignment with high accuracy and high throughput. Nucleic Acids Res. 32, 1792–1797. doi: 10.1093/nar/gkh340 15034147 PMC390337

[B13] Fedoroff N. V. Battisti D. S. Beachy R. N. Cooper P. J. Fischhoff D. A. Hodges C. N.. (2010). Radically rethinking agriculture for the 21st century. Science 327, 833–834. doi: 10.1126/science.1186834 20150494 PMC3137512

[B14] Ferreira K. N. Iverson T. M. Maghlaoui K. Barber J. Iwata S. (2004). Architecture of the photosynthetic oxygen-evolving center. Science 303, 1831–1838. doi: 10.1126/science.1093087 14764885

[B15] Gadjev I. Vanderauwera S. Gechev T. S. Laloi C. Minkov I. N. Shulaev V.. (2006). Transcriptomic footprints disclose specificity of reactive oxygen species signaling in arabidopsis. Plant Physiol. 141, 436–445. doi: 10.1104/pp.106.078717 16603662 PMC1475436

[B16] Gasch A. P. Eisen M. B. (2002). Exploring the conditional coregulation of yeast gene expression through fuzzy k-means clustering. Genome Biol. 3, 1–22. doi: 10.1186/gb-2002-3-11-research0059 PMC133443 12429058

[B17] Gill S. S. Tuteja N. (2010). Reactive oxygen species and antioxidant machinery in abiotic stress tolerance in crop plants. Plant Physiol. Biochem. 48, 909–930. doi: 10.1016/j.plaphy.2010.08.016 20870416

[B18] Hartman S. Liu Z. Van Veen H. Vicente J. Reinen E. Martopawiro S.. (2019). Ethylene-mediated nitric oxide depletion pre-adapts plants to hypoxia stress. Nat. Commun. 10, 4020. doi: 10.1038/s41467-019-12045-4 31488841 PMC6728379

[B20] He M. He C. Q. Ding N. Z. (2018b). Abiotic stresses: General defenses of land plants and chances for engineering multistress tolerance. Front. Plant Sci. 9, 1771. doi: 10.3389/fpls.2018.01771 30581446 PMC6292871

[B19] He H. Van Breusegem F. Mhamdi A. (2018a). Redox-dependent control of nuclear transcription in plants. J. Exp. Bot. 69, 3359–3372. doi: 10.1093/jxb/ery130 29659979

[B21] Huang H. Ullah F. Zhou D. X. Yi M. Zhao Y. (2019). Mechanisms of ROS regulation of plant development and stress responses. Front. Plant Sci. 10, 800. doi: 10.3389/fpls.2019.00800 31293607 PMC6603150

[B22] Huang S. Van Aken O. Schwarzlander M. Belt K. Millar A. H. (2016). The roles of mitochondrial reactive oxygen species in cellular signaling and stress response in plants. Plant Physiol. 171, 1551–1559. doi: 10.1104/pp.16.00166 27021189 PMC4936549

[B31] Jones M. R. Fyfe P. K. (2001). Photosynthesis new light on biological oxygen production. Curr. Biol. 11 (8):R318–R321. doi: 10.1016/s0960-9822(01)00174-9 11369223

[B23] Kanwar P. Baby D. Bauer P. (2021). Interconnection of iron and osmotic stress signalling in plants: is FIT a regulatory hub to cross-connect abscisic acid responses? Plant Biol. (Stuttg) 23 Suppl 1, 31–38. doi: 10.1111/plb.13261 33772999

[B44] Karuppanapandian T. Moon J.-C. Kim C. Kim W. (2011). Reactive oxygen species in plants their generation, signal transduction, and scavenging mechanisms. Aust. J. Crop Sci. 5 (6), 709–725. doi: 10.1111/j.1365-3040.2009.02041.x

[B24] Kim D. Langmead B. Salzberg S. L. (2015). HISAT: a fast spliced aligner with low memory requirements. Nat. Methods 12, 357–360. doi: 10.1038/nmeth.3317 25751142 PMC4655817

[B25] Kim S. Park M. Yeom S. I. Kim Y. M. Lee J. M. Lee H. A.. (2014). Genome sequence of the hot pepper provides insights into the evolution of pungency in capsicum species. Nat. Genet. 46, 270–278. doi: 10.1038/ng.2877 24441736

[B26] Klopfenstein D. V. Zhang L. Pedersen B. S. Ramirez F. Warwick Vesztrocy A. Naldi A.. (2018). GOATOOLS: A Python library for gene ontology analyses. Sci. Rep. 8, 10872. doi: 10.1038/s41598-018-28948-z 30022098 PMC6052049

[B27] Letunic I. Bork P. (2016). Interactive tree of life (iTOL) v3: an online tool for the display and annotation of phylogenetic and other trees. Nucleic Acids Res. 44, W242–W245. doi: 10.1093/nar/gkw290 27095192 PMC4987883

[B28] Lin G. Chai J. Yuan S. Mai C. Cai L. Murphy R. W.. (2016). VennPainter: A tool for the comparison and identification of candidate genes based on Venn diagrams. PloS One 11, e0154315. doi: 10.1371/journal.pone.0154315 27120465 PMC4847855

[B29] Liu F. Yu H. Deng Y. Zheng J. Liu M. Ou L.. (2017). PepperHub, an informatics hub for the chili pepper research community. Mol. Plant 10, 1129–1132. doi: 10.1016/j.molp.2017.03.005 28343897

[B30] Love M. I. Huber W. Anders S. (2014). Moderated estimation of fold change and dispersion for RNA-seq data with DESeq2. Genome Biol. 15, 550. doi: 10.1186/s13059-014-0550-8 25516281 PMC4302049

[B32] Miller G. Suzuki N. Ciftci-Yilmaz S. Mittler R. (2010). Reactive oxygen species homeostasis and signalling during drought and salinity stresses. Plant Cell Environ. 33 (4), 453–467. doi: 10.1111/j.1365-3040.2009.02041.x 19712065

[B33] Nazir F. Hussain A. Fariduddin Q. (2019). Hydrogen peroxide modulate photosynthesis and antioxidant systems in tomato (Solanum lycopersicum l.) plants under copper stress. Chemosphere 230, 544–558. doi: 10.1016/j.chemosphere.2019.05.001 31125883

[B34] Qin C. Yu C. Shen Y. Fang X. Chen L. Min J.. (2014). Whole-genome sequencing of cultivated and wild peppers provides insights into capsicum domestication and specialization. Proc. Natl. Acad. Sci. U.S.A. 111, 5135–5140. doi: 10.1073/pnas.1400975111 24591624 PMC3986200

[B35] Rodriguez M. C. Petersen M. Mundy J. (2010). Mitogen-activated protein kinase signaling in plants. Annu. Rev. Plant Biol. 61, 621–649. doi: 10.1146/annurev-arplant-042809-112252 20441529

[B36] Sachdev S. Ansari S. A. Ansari M. I. Fujita M. Hasanuzzaman M. (2021). Abiotic stress and reactive oxygen species: Generation, signaling, and defense mechanisms. Antioxidants (Basel) 10 (2), 277. doi: 10.3390/antiox10020277 33670123 PMC7916865

[B37] Sandalio L. M. Romero-Puertas M. C. (2015). Peroxisomes sense and respond to environmental cues by regulating ROS and RNS signalling networks. Ann. Bot. 116, 475–485. doi: 10.1093/aob/mcv074 26070643 PMC4577995

[B38] Sarvajeet Singh Gill Khan N. A. Anjum N. A. Tuteja N. (2011). Amelioration of cadmium stress in crop plants by nutrient management: Morphological, physiological and biochemical aspects. Plant Stress 5 (1), 1–23. doi: 10.4161/psb.6.2.14880

[B39] Seok H. Y. Tran H. T. Lee S. Y. Moon Y. H. (2022). AtERF71/HRE2, an arabidopsis AP2/ERF transcription factor gene, contains both positive and negative cis-regulatory elements in its promoter region involved in hypoxia and salt stress responses. Int. J. Mol. Sci. 23 (10), 5310. doi: 10.3390/ijms23105310 35628120 PMC9140466

[B40] Smit M. E. Mcgregor S. R. Sun H. Gough C. Bagman A. M. Soyars C. L.. (2020). A PXY-mediated transcriptional network integrates signaling mechanisms to control vascular development in arabidopsis. Plant Cell 32, 319–335. doi: 10.1105/tpc.19.00562 31806676 PMC7008486

[B41] Song H. Chen F. Wu X. Hu M. Geng Q. Ye M.. (2022). MNB1 gene is involved in regulating the iron-deficiency stress response in arabidopsis thaliana. BMC Plant Biol. 22, 151. doi: 10.1186/s12870-022-03553-5 35346040 PMC8961904

[B42] Sun Y. Zhang C. Qi A. Luo W. (2009). “Effects of hydrogen peroxide on the germination characteristics of cucumber seeds”. in International Conference on Environmental Science and Information Application Technology. (IEEE), 272–275.

[B43] Tang H. Bi H. Liu B. Lou S. Song Y. Tong S.. (2021). WRKY33 interacts with WRKY12 protein to up-regulate RAP2.2 during submergence induced hypoxia response in arabidopsis thaliana. New Phytol. 229, 106–125. doi: 10.1111/nph.17020 33098101

[B45] Tomato Genome C. (2012). The tomato genome sequence provides insights into fleshy fruit evolution. Nature 485, 635–641. doi: 10.1038/nature11119 22660326 PMC3378239

[B47] Van Nguyen T. Park C. R. Lee K. H. Lee S. Kim C. S. (2021). BES1/BZR1 homolog 3 cooperates with E3 ligase AtRZF1 to regulate osmotic stress and brassinosteroid responses in arabidopsis. J. Exp. Bot. 72, 636–653. doi: 10.1093/jxb/eraa458 33529338

[B52] Wang Z.-Y. Gendron J. Nakano T. He J. Chen M. Vafeados D.. (2002). Nuclear-localized BZR1 mediates brassinosteroid-induced growth and feedback suppression of brassinosteroid biosynthesis. Developmental Cell 2 (4), 505–513. doi: 10.1016/S1534-5807(02)00153-3 11970900

[B48] Willems P. Mhamdi A. Stael S. Storme V. Kerchev P. Noctor G.. (2016). The ROS wheel: Refining ROS transcriptional footprints. Plant Physiol. 171, 1720–1733. doi: 10.1104/pp.16.00420 27246095 PMC4936575

[B49] Xiong J. Yang Y. Fu G. Tao L. (2015). Novel roles of hydrogen peroxide (H(2)O(2)) in regulating pectin synthesis and demethylesterification in the cell wall of rice (Oryza sativa) root tips. New Phytol. 206, 118–126. doi: 10.1111/nph.13285 25615266

[B50] Yin Y. Vafeados D. Tao Y. Yoshida S. Asami T. Chory J. (2005). A new class of transcription factors mediates brassinosteroid-regulated gene expression in arabidopsis. Cell 120, 249–259. doi: 10.1016/j.cell.2004.11.044 15680330

[B51] Yu G. Wang L. G. Han Y. He Q. Y. (2012). clusterProfiler: An r package for comparing biological themes among gene clusters. OMICS 16, 284–287. doi: 10.1089/omi.2011.0118 22455463 PMC3339379

[B53] Zhu J. K. (2016). Abiotic stress signaling and responses in plants. Cell 167, 313–324. doi: 10.1016/j.cell.2016.08.029 27716505 PMC5104190

[B54] Zubo Y. O. Blakley I. C. Franco-Zorrilla J. M. Yamburenko M. V. Solano R. Kieber J. J.. (2018). Coordination of chloroplast development through the action of the GNC and GLK transcription factor families. Plant Physiol. 178, 130–147. doi: 10.1104/pp.18.00414 30002259 PMC6130010

